# Supplementary material for: All-at-once RNA folding with 3D motif prediction framed by evolutionary information
Source: Nat Methods. 2025 Oct 3;22(10):2094–106. doi: 10.1038/s41592-025-02833-w (PMC12510878; doi:10.1038/s41592-025-02833-w)
Supplement: Supplementary file 3 — Source code to run CaCoFold-R3D. Data and results. [file 41592_2025_2833_MOESM3_ESM.gz › supplemental_material/results/CaCoFold-R3D/CaCoFold-R3D_Rfam_vshuffle/RF01177_snR67.R2R.sto.pdf]

# RF01177_snR67

5′-Y ● ● ● R U G A U G A C ● ● G Y U G U C G C C C C A A R C G G A ● ● Y ● ● R ● R A U G A C Y G A Y U Y ● A Y ● ● R ● ● R ○ ○ ● A A A C A C U G A A A A U U C U G A ● ●
